# Ophthalmology and Ophthalmoscopy, for Practitioners and Students of Medicine

**Published:** 1889-11

**Authors:** 


**Ophthalmology and Ophthalmoscopy, for Practitioners and Students of Medicine.** By Dr. HERMANN SCHMIDT-RIMPLER, Professor of Ophthalmology, and Director of the Ophthalmological Clinic in Marburg, Germany. Edited by D. B. ST. JOHN ROOSA, M. D., LL. D. New York: Wm. Wood & Co., 56 and 58 Lafayette place. Pp. 571.

It is with some degree of pleasure that the reviewer is brought face to face with the English translation of Schmidt-Rimpler's excellent treatise on Ophthalmology. Having many times been obliged to consult it as a text-book during his career in the German universities, where it ranks as highest authority, he is able to testify to its great merit.

The affiliation of D. B. St. John Roosa, of New York, to this work, as editor, bespeaks for it a scientific worth of the highest order. In its native tongue, it has passed through two large editions; this, the third, has been subjected to careful revision, and changed in accordance with the constant increase of ophthalmological study. The author divides his treatise into four parts.

Part I. treats of General Observations on the Examination and Treatment of the Eye, Errors of Refraction and Accommodation, Amblyopia, and Amaurosis. This part of the work has been revised but little, and contains few annotations from its American editor. The laws of Optics, as applied to accommodation and refraction of the human eye, are lucidly described, and are accompanied by many excellent illustrations.

Part II. treats of Ophthalmoscopy, Ophthalmoscopic Appearances in the Healthy Eye, and Diseases of the Optic Nerve, Retina, Choroid, and Vitrous Body.

The Diseases of the Internal Eye are accompanied by three full-page colored plates, containing eighteen representations of the most common affections of the fundus.

Part III. is devoted to the discussion of Glaucoma, Diseases of the Lens, Conjunctiva, Cornea, Sclera, Iris, Ciliary Body, and Sympathetic Affections.

Author and editor disagree in the use of weak solutions of nitrate of silver in the new-born. The author encourages the practice, so general in Germany, of instilling into the eyes of all new-born a drop of two-per-cent. solution of nitrate of silver, as prophylaxis against Blenorrhea neonatorum. The editor advises against the use of nitrate of silver, in any form, in any of the earlier periods of this disease.

Part IV. treats of Diseases of the Ocular Muscles, Orbits, Eyelids, and Lachrymal Apparatus.

Numerous admirable illustrations adorn this and the preceding parts of the work, many of which are taken from Stellwag's treatise.

The typography of the work is excellent, and is in keeping with the other works on "Specialties in the Practice of Medicine."

